# Bipolar Resistive Switching Behavior of PVP-GQD/HfOx/ITO/Graphene Hybrid Flexible Resistive Random Access Memory

**DOI:** 10.3390/molecules26226758

**Published:** 2021-11-09

**Authors:** Jin Mo Kim, Sung Won Hwang

**Affiliations:** 1 Micro LED Research Center, Korea Photonics Technology Institute, Gwangju 61007, Korea; jmkim@kopti.re.kr; 2 Department of System Semiconductor Engineering, Sangmyung University, Cheonan 31066, Korea

**Keywords:** HfOx, GQD, RRAM, memristive devices, resistive switching

## Abstract

We have investigated highly flexible memristive devices using reduced graphene oxide (RGO) nanosheet nanocomposites with an embedded GQD Layer. Resistive switching behavior of poly (4-vinylphenol):graphene quantum dot (PVP:GQD) composite and HfOx hybrid bilayer was explored for developing flexible resistive random access memory (RRAM) devices. A composite active layer was designed based on graphene quantum dots, which is a low-dimensional structure, and a heterogeneous active layer of graphene quantum dots was applied to the interfacial defect structure to overcome the limitations. Increasing to 0.3–0.6 wt % PVP-GQD, V_f_ changed from 2.27–2.74 V. When negative deflection is applied to the lower electrode, electrons travel through the HfOx/ITO interface. In addition, as the PVP-GQD concentration increased, the depth of the interfacial defect decreased, and confirmed the repetition of appropriate electrical properties through Al and HfOx/ITO. The low interfacial defects help electrophoresis of Al^+^ ions to the PVP GQD layer and the HfOx thin film. A local electric field increase occurred, resulting in the breakage of the conductive filament in the defect.

## 1. Introduction

Changes in the capacity and intensive industrial structure of information storage devices require high-performance storage devices for the functionality of complex systems [1,2,3,4,5,6,7,8,9,10,11,12]. In particular, in the case of Resistive Random Access Memories (RRAMs), various studies on organic/inorganic structures have been conducted to maximize the price and performance of existing memory semiconductors in terms of integration, low power, and fairness compared to simple structures [13,14,15,16,17,18]. For example, not only traditional inorganic structures such as aluminum oxide film and titanium dioxide, but also organic structures such as poly(methyl-me-thacrylate) [19], polyvinyl alcohol [20], poly(4-vinylphenol) (PVP), graphene oxide film, etc. Studies on two-dimensional structures are also being focused. However, in the stacked structure of organic field effect transistors (OFETs) devices and various inorganic thin films, it is impossible to control defects fundamentally occurring at the interface, which directly deteriorated device performance. Among these materials, graphene oxide (GO) has been extensively studied to develop electronic devices, and reduced graphene oxide (rGO) can be produced from electrically insulating GO by removing the oxygen groups via thermal and chemical reductions. Therefore, GO thin films can be used as a promising active layer in resistive switching memories. The microscopic origin of the resistive switching phenomenon in GO-based memories has been generally explained by oxygen ion migration and the formation of a metal filament within GO films. In this study, a low-dimensional graphene quantum dot-based composite active layer was designed, applying a heterogeneous active layer of graphene quantum dots to the interfacial defect structure. To present the performance degradation issue, a bipolar resistive switching memory device of Al/PVP GQD/HfOx/ITO structure through bias change was fabricated, and characteristics were checked. The high-performance repeatability according to the set and reset confirmed voltage changes in individual devices. The resistive switching operation according to the concentration of active layers was guaranteed. In addition, as the concentration of PVP GQDs increased, the defect depth at the interface decreased, confirming the appropriate electrical repeat characteristics at the voltage change through the Al and HfOx/ITO electrodes. This work first provides evidence of localized graphitic channels formed in nanoscale GO thin films by oxygen ion diffusion.

## 2. Materials and Methods

### 2.1. Materials

The following processes fabricated GQDs. Graphene oxide (GO) was prepared from graphite powder using the Hummers method [21,22]. The GO was deoxidized in a horizontal furnace at 350–450 °C for four hours under Ar to produce reduced graphene oxide powder. About 5.0–6.5 g of graphene oxide powder was oxidized by ultrasonication in concentrated 15 mL H_2_SO_4_ and 40 mL HNO_3_ for 22 h. In addition, 350 mL deionized water was used to dilute the mixture. A 200 nm membrane filtered it to neutralize. The size-reduced and purified 2.5 g GO powder was redissolved in 15 mL deionized water, and NaOH was used to set the pH to 7.8. The aliquot was transferred to a nitrogen-ambient furnace and heated at 350–400 °C for six hours. After cooling to room temperature, the resulting powder was redispersed in 40-mL DI water for two hours under ultrasonication. Then, by filtering the resulting suspension through a 200-nm nanoporous membrane, a brown solution was separated. Since the colloidal solution still contained some large graphene nanoparticles (<200 nm) emitting weak blue fluorescence, it was further filtered in a dialysis bag with a cutoff of 3500 Da molecular weight overnight, thereby producing strongly fluorescent GQDs. The GQDs were separated into different sizes using several dialysis bags of 1000–50,000 Da and a 20-nm nanoporous membrane.

### 2.2. Device Fabrication

After the direct synthesis of the extracted GQD and PVP, graphene quantum dots of each shape were directly grown on a patterned transparent electrode in a Cu thin film through a modified CVD method. After washing the composite GQD layer through the deionized water cleaning process, heat treatment was performed. The solution for dielectric structure of PVP was prepared by sonication of 1.7–5.8 wt % PVP in 2-propanol for 60 min for preparing five samples with different PVP concentrations. The solution was spin coated at 6500 rpm for 45 s to obtain a ~75 nm thick PVP layer. A cross-bar type Al/PVP GQD/HfOx structure was deposited on an ITO substrate by thermal evaporation using a 150 µm wide overlapping hard mask. The Al electrode was fabricated using the electron beam deposition method, and the HfOx structure was deposited on the Ti adhesive layer.

### 2.3. Characterization

The morphologies of GQDs were analyzed using an HRTEM (High-resolution Transmission Microscopy) (FEI Tecnai F30 S-Twin). To make the HRTEM specimens, the GQDs were dispersed in DI water, a drop of which was then put on a C- or SiO-coated Cu grid (Tedpella, Inc., Redding, CA, USA) and mica substrate, respectively. The current-voltage (I–V) curve of the device was analyzed in an electrical and electronic workstation (Keithley 2400).

## 3. Results and Discussion

**Figure 1:**
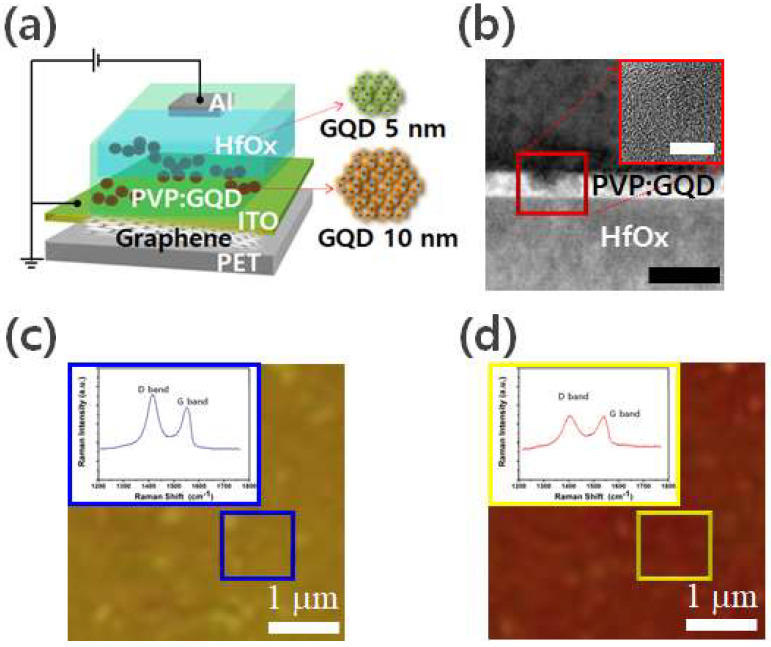
Schematic illustration (**a**) of the device architecture based on PVP GQD/HfOx/ITO/graphene. (**b**) and TEM image of PVP GQD/HfOx layer. The scale bar indicates 20 nm. Inset on right panel: high-resolution TEM image showing GQDs. Scale bar, 5 nm. Characterization of chemical state in the GQD layers, the ON and the OFF state. Raman intensity ratio maps of D and G bands at (**c**) ON state, and (**d**) OFF state. Inset: Raman profiles of the blue and yellow box in (**c**,**d**).

Figure 1a is a schematic diagram applied to the Poly(4-vinylphenol) (PVP)/HfOx stack structure on the ITO substrate. To fabricate a resistive memory structure, an ultra-thin HfOx layer was synthesized by atomic layer deposition on an ITO transparent substrate, which is the lower electrode, and graphene quantum dots in PVP were synthesized. A conductive Al ion structure was applied. After the 10 nm or smaller graphene quantum dot structure was fabricated, Al ions were formed as channels in the graphene quantum dot region [23,24]. Figure 1b is a cross-sectional bright-field TEM image in the ON state obtained after applying a negative bias to the upper electrode. The non-uniform interface state was confirmed in the Al/PVP GQD/HfOx/ITO stack structure. Although the graphene quantum dot region in PVP has a more regular and densely stacked path than the off state, vacancy in the metal atomic layer increased at the interface, which means that the defects in the interfacial region in Al and HfOx increased. As shown in the TEM image, in the Al/PVP GQD/HfOx/ITO structure, locally bonded atomic layer regions were observed at the heterogeneous interface, and it can be judged that the Al oxide film affected the charge transport layer formation in the ON state. An irregular interfacial structure appeared in another localized region, and the boundary between the graphene quantum dot channel crystallization region and the HfOx region could be identified.

It is not easy to create a controllable electric field in a specific region because a typical HfOx thin film has defects related to interfacial oxygen ions. The highly crystallized graphene quantum dot region in PVP, considered as a conductive channel, makes the inter-interface defect spacing large by the change in the desorption of Al ions and HfOx oxygen ions, but by the movement of the metal interface and HfOx oxygen ions under a strong electric field to the conductive PVP graph. It can be confirmed that the graphene quantum dot channel is formed gradually [25,26,27,28]. Figure 1c,d show the Raman spectra of the GQDs for chemical state in the GQD layers, the ON and the OFF state and PVP GQD/HfOx/ITO/graphene substrate. The Raman spectrum of the GQD resolves into two distinctive D and G bands at ∼1410 cm^−1^ and ∼1550 cm^−1^, respectively. The D and G bands are almost not shifted by varying the ON and the OFF state. In contrast, the G band shows a considerable GQD edge dependent shift. There have been numerous studies on the behaviors of Raman bands in graphene sheets. The G band corresponds to the E_2g_ phonon at the Brillouin zone center, and its frequency is known to downshift as strain increases [29].

**Figure 2:**
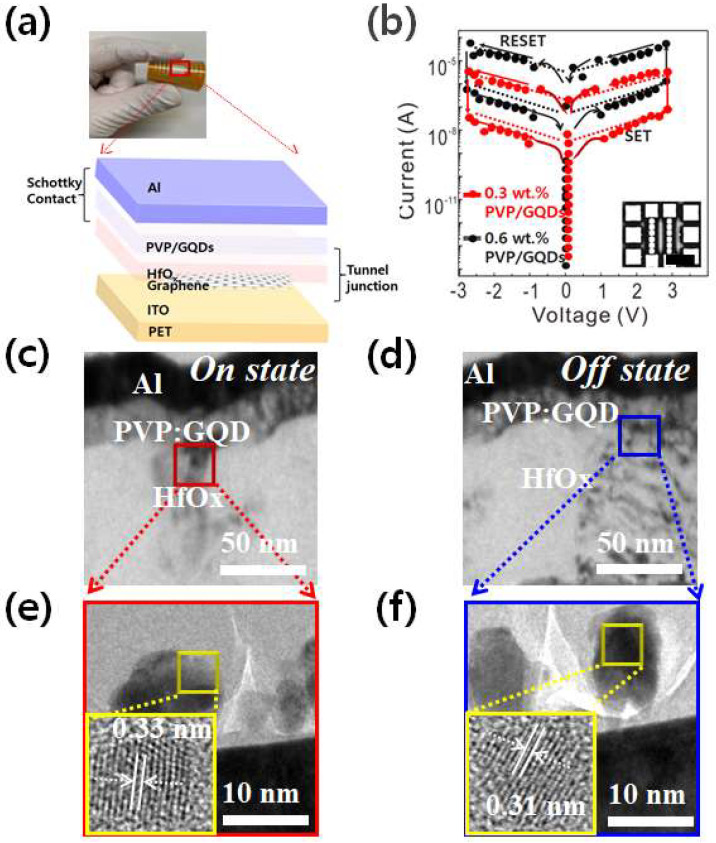
(**a**) Schematic illustrating the flexible device structure with PVP GQD layer; (**b**) I–V curves of Al/PVP GQD/HfOx/ITO/graphene/PET memory devices plotted on a semi-logarithmic scale. The inset is an optical microscopy image of Al/PVP GQD/HfOx/ITO/graphene device (scale bar: 200 μm). Cross-sectional TEM images of (**c**) conductive channel region at the ON state and (**d**) the OFF state; (**e**,**f**) corresponding cross-sectional HR-TEM images obtained in the red and blue rectangular region of (**c**,**d**), respectively.

Figure 2a is a current–voltage (I–V) curve of the PVP-GQD/HfOx/ITO structure. A negative bias was applied to the upper electrode, keeping the lower electrode at ground potential. A compliance current of 10 mA was applied to protect the device. The PVP GQD/HfOx/ITO structure showed stable resistive switching operation without structural deformation of the upper electrode. The initial switching occurred at Vf = 1.84 V for 0.5 wt % PVP, and Vf changed from 2.27 to 2.74 V when increasing from 1.5 to 3.5 wt % PVP. When changed up the sweep voltage to 10 V, there was no resistive switching characteristic at 4.0 wt % PVP. In the next switching stage, at 0.5 wt % PVP, the high resistance state (HRS) was maintained when the bias was applied below 0.5 V, and when the bias was applied over 1.14 V, the current rapidly increased, and the low resistance state (LRS) was converted [30,31].

The applied bias was continuously converted to a conductive high resistance state until it was swept to V reset = −0.47 V. On the other hand, in the 0.5 ~ 1.5 wt % RRAM device, the resistive switching behavior of set and reset was confirmed at V set = 1.53 V and V reset = −0.76 V. It was found that it was difficult to define the characteristics for the switching characteristics of the 3.5–4.0 wt % device due to the low voltage switching operation due to interfacial defects. At 0.18 V, the Ion/Ioff ratio is more than 68, indicating an evident bipolar resistive switching characteristic. The resistance of repeated V set/V reset and stable repetitive operation of 100 DC cycles made it possible to confirm the uniform switching voltage characteristics. Figure 2b shows the most consistent number of processes and resistance switching results in 10 or more devices randomly selected to evaluate whether there is a significant difference between devices and the switching operation characteristics. It was confirmed that there was no deterioration in the device’s constant data retention characteristics and current change due to the bias of V = 0.23 V in the low-resistance state and V = −0.21 V in the high-resistance state. Figure 2c–f show an HRTEM image of the Al/PVP GQD/HfOx layer at the ON and the OFF state. The inset in Figure 2e,f describes a HR-TEM images obtained in the red and blue rectangular region of (c) and (d), respectively. The observation is that GQD is organized into a PVP layer as nanoparticles that uniformly incorporate the interface of HfOx at the ON state, meaning that the crystallinity of the GQD layer increased. In contrast, the GQD is activated into PVP layer as aggregation that nonuniformly penetrate the interface of HfOx at the OFF state.

**Figure 3:**
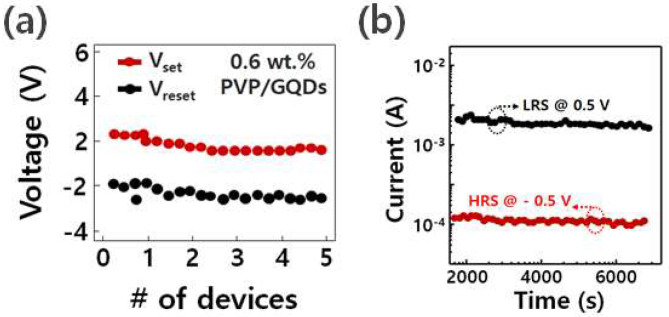
(**a**) Device-to-device variation of the switching; (**b**) a plot of retention time measurement by applying voltages of −0.5 V and 0.5 V in HRS and LRS, respectively.

Figure 3a shows the high-resistance (HRS) state for the ohmic conductive region at high bias voltage to understand the conduction mechanism in the current–voltage characteristics. Ohmic conduction is a characteristic that occurs due to moving electrons in the conduction band and the movement of holes in the valence band. This reflects the characteristics of charge mobility depending on the upper and lower electrode states, total carrier density, and material dielectric constant. In addition, in the set state of the device, an ohmic characteristic showing the metal behavior of the conductive filament in the low resistance region was exhibited. After applying a bias to the electrode, it is known that, under the influence of an extremely high electric field, oxygen vacancy, which serves as a trap site for the injected charge from the lower electrode, is created in the metal oxide film [32].

These oxygen vacancies can be considered as pervasive point defects in metal oxides. The atomic bonds are weakened when a changing electric field is applied to the HfOx layer, which is particularly important for controlling resistive memory properties, available physical properties [33]. Figure 3b shows the data retention time by an applied voltage of −0.5 V to 0.5 V in the high resistance (HRS) and low resistance (LRS) states. The data retention time characteristics of 7000 s in each state were exhibited without deteriorating the current level.

**Figure 4:**
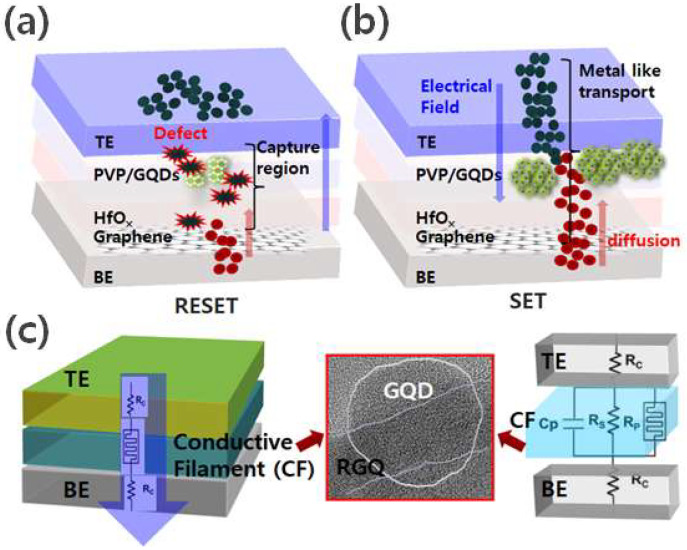
(**a**) Schematic illustrating the device structure with an atomic defect in PVP GQD layer; (**b**) oxidation of Al to Al^+^ ions and diffusion of Al^+^ ions into PVP GQD layer, reduction of Al^+^ ions. (**c**) Schematic of the conductive filament (CF) evolution model in the SET process and equivalent circuit with parasitic effects.

Figure 4a,b are schematic diagrams illustrating the resistive switching mechanism of Al/PVP-GQD/HfOx/ITO/graphene devices. In several research groups, the formation of conductive filaments has been described as influenced by electron transfer of Al^+^ ions in HfOx thin films and PVP GQD layers [34]. When a positive bias is applied to the upper electrode, the oxidation of Al atoms occurs. As a result, Al^+^ ions are generated, which electromigrates through the PVP GQD, and when a negative bias is applied to the lower electrode, the HfOx/ITO interface electrons move through. While interacting with electrons injected from the lower electrode, Al^+^ ions decreased and switched the device to a low-resistance state. Under a high electric field, the electron mobility of Al^+^ ions is significant where deep defects are located, and the electron mobility of Al^+^ ions is considerable in the defects occurring at the lower electrode and oxide film interface [35]. During the reset process with the change in polarity due to the reverse bias, the electric field and current density were maximized near the interfacial defect, and the electrochemical reaction and destruction of the conductive filament by joule heat occurred.

Conductive filaments with a diameter of several to several tens of nanometers generated local joule heat, and a current of 17 mA or more flowed through it, which was sufficient to induce a high current density of 10^10^ A/cm^2^ or more as a result. As observed in previous research reports, the conventional resistive switch metal thin film conductive filament formed an irregular shape, and the reliability was reduced due to the increase of irregular interfacial defects during the reset process [36]. Figure 4c shows schematic diagrams illustrating the resistive switching mechanism of the conductive filament (CF) evolution model in the SET process and equivalent circuit with parasitic effects. The CF formation is assisted by electro-migration of Al^+^ ions in PVP/GQD layer and HfOx/graphene layer. The Al^+^ ions get reduced to Al atoms, the CF is formed, and device is switched to LRS. The detailed analysis of HRTEM images for GQDs shows that the periphery of GQDs consists of mixed zigzag and armchair edges. The equivalent circuit of RRAM is shown in the inset. It consists of contact resistance (Rc), a parallel resistance (Rp), a parallel capacitance (Cp), and the resistive switching elements (Rs).

## 4. Conclusions

In this study, the resistive switching behavior and electrical characteristics of flexible devices with Al/PVP GQD/HfOx/ITO/graphene/PET structures were presented according to the GQD concentration in the PVP GQD active layer. As observed in the current–voltage characteristics, the reset voltage decreased as the concentration of the active layer, including GQDs increasing. This result is because the defect depth at the interface decreased as the PVP GQD concentration increased. Appropriate electrical repeatability was observed in the voltage change through Al and HfOx/ITO/graphene electrodes, and this result can be presented as a highly reliable bipolar resistive switching operation. The low voltage and stable switching operation reduced the interfacial defects in the device structure, and the electromigration of Al^+^ ions through the PVP GQD layer and the HfOx thin film was supported. Reversing the bias voltage resulted in high current density and local electric field increasing at the junction due to electrochemical interaction and joule heating. These results indicated that the conductive filament was broken at the pinhole tip. In addition, GQD channels and conductive filament pathways instead of multiple conductive filament pathways at the PVP/GQD interface helped charge transfer, which was shown to affect the performance of the resistive switching device.

## Data Availability

The data presented in this study are available on request from the corresponding author.

## Sample Availability

Samples of the compounds are not available from the authors.
